# Underutilisation of cardiovascular medications among at-risk individuals

**DOI:** 10.1111/j.1742-1241.2009.02258.x

**Published:** 2010-04

**Authors:** S J Lewis, J G Robinson, K M Fox, S Grandy, for the SHIELD Study Group

**Affiliations:** 1 Northwest Cardiovascular InstitutePortland, OR, USA; 2 University of IowaIowa City, IA, USA; 3 Strategic Healthcare SolutionsLLC, Monkton, MD, USA; 4 AstraZeneca Pharmaceuticals LPWilmington, DE, USA

## Abstract

**Aims:**

Guidelines recommend antihypertensive, lipid-lowering and/or antiplatelet therapy for prevention of cardiovascular disease (CVD). This study examined the utilisation of cardiovascular therapies among individuals at CVD risk to assess adherence to guidelines.

**Methods:**

Respondents to the SHIELD study were classified based on National Cholesterol Education Program Adult Treatment Panel III risk categories. High coronary heart disease (CHD) risk (*n* = 7510) was defined as self-reported diagnosis of heart disease/heart attack, narrow or blocked arteries, stroke or diabetes; moderate risk (*n* = 4823) included respondents with ≥ 2 risk factors (i.e., men > 45 years, women > 55 years, hypertension, low high-density lipoprotein cholesterol, smoking and family history of CHD); and low risk (*n* = 5307) was 0–1 risk factor. Respondents reporting a myocardial infarction, stroke or revascularisation at baseline (prior CVD event) (*n* = 3777), those reporting a new CVD event during 2 years of follow up (*n* = 953), and those with type 2 diabetes mellitus (*n* = 3937) were evaluated. The proportion of respondents reporting treatment with lipid-lowering, antiplatelet or antihypertensive agents was calculated.

**Results:**

Utilisation of lipid-lowering therapy was low (≤ 25%) in each group. Prescription antithrombotic therapy was minimal among respondents with prior CVD events, but 47% received antihypertensive medication. No use before or after a new CVD event was reported by 36% of respondents for lipid-lowering, 32% for antithrombotic and > 50% for antihypertensive medications.

**Conclusions:**

More than 50% of at-risk respondents and > 33% of respondents with new CVD events were not taking CVD therapy as recommended by guidelines.

**What’s known**

Cardiovascular disease is a prevalent condition that is the leading cause of death in the United States and several national guidelines provide recommendations for the treatment and prevention of cardiovascular disease in routine clinical practice.

**What’s new**

This study highlights the gap in the utilisation of cardiovascular drug therapies, including statins, antiplatelet/anticoagulant and anti-hypertensive agents among respondents with high and moderate coronary heart disease risk and those with a prior cardiovascular event or new incident event. The findings indicate that the treatment guidelines have not been translated into clinical practice for many individuals at risk of cardiovascular disease.

## Introduction

Cardiovascular disease (CVD), including ischaemic coronary heart disease (CHD), stroke and peripheral vascular disease, is the leading cause of death in the United States (1). More than 1 million Americans die each year from myocardial infarction (MI) and other forms of CHD (1). Various national scientific guidelines recommend specific pharmacotherapies for the treatment and prevention of CVD (2–4).

The American Heart Association (AHA)/American College of Cardiology (ACC) guidelines (2) for secondary prevention in patients with coronary and other atherosclerotic vascular disease recommended the following therapies: (i) angiotensin-converting enzyme inhibitor (ACEI) for all patients with CVD and ejection fraction < 40% and those with hypertension, diabetes or chronic kidney disease, unless contraindicated, (ii) angiotensin II receptor blocker (ARB) for those intolerant of ACEIs and who have heart failure or MI with ejection fraction ≤ 40%, (iii) beta-blocker for those who have MI or acute coronary syndrome, (iv) antiplatelet or anticoagulant therapy for those who have acute coronary syndrome, percutaneous coronary intervention, or MI, and aspirin for all patients unless contraindicated, and (v) lipid-lowering drug therapy if low-density lipoprotein cholesterol (LDL-C) is ≥ 100 mg/dl. The AHA 2004 guidelines for CVD prevention in women, additionally recommended aspirin use for moderate- and high-risk women (3). The National Cholesterol Education Program Adult Treatment Panel III (NCEP ATP III) guidelines recommend statin therapy for individuals at high or moderate CHD risk if their LDL-C is not at target goal (4).

The purpose of this study was to evaluate the utilisation of prescription therapies and aspirin among a large, community-based cohort of individuals at risk for CVD, including those with type 2 diabetes mellitus (T2DM) or prior CVD events (MI, stroke), or who experienced a new CVD event during follow up to assess whether prescribing guidelines were being adopted.

## Methods

Individuals at risk for or with a prior history of CVD events (i.e. MI, stroke or revascularisation) were identified from the **S**tudy to **H**elp **I**mprove **E**arly evaluation and management of risk factors **L**eading to **D**iabetes (SHIELD). SHIELD is a population-based survey conducted to better understand the risk and disease burden of diabetes and CVD. SHIELD included an initial screening phase to identify cases of interest in the general population and a detailed baseline survey to follow up identified cases for health status, health knowledge, attitudes, behaviours and treatment. Annual follow-up surveys were administered to obtain information about changes in health status, behaviours and treatment. A detailed description of the SHIELD methodology has been published previously (5,6).

In brief, the screening survey was mailed to a stratified random sample of 200,000 US households, representative of the US population for geographical residence, household size and income and age of head of household (7). The head of household provided responses for up to four adult (aged ≥ 18 years) household members, resulting in a response rate of 63.7% (127,420 households for 211,097 adults). The baseline survey was sent to 22,001 selected individuals derived from the screening respondents. A response rate of 71.8% was obtained (*n* = 15,794).

In August 2005, the first annual follow-up survey was mailed to all individuals selected for the baseline survey who were still enrolled in the household panel (*n* = 19,613). The second follow-up survey was mailed in July 2006 to individuals who had returned either or both the baseline and first annual questionnaires (*n* = 18,445). The 2005 survey had a response rate of 72%, and a 75% response rate was obtained for the 2006 survey (*n* = 13,877). This study utilised the baseline, 2005 and 2006 survey responses. De-identified information was analysed in compliance with the Health Insurance Portability and Accountability Act. SHIELD utilised a household consumer panel, and as consumer panels are not considered clinical studies, institutional review board approval was not required.

### Study population

Three primary respondent groups were identified from the baseline survey: (i) high CHD risk, (ii) moderate CHD risk, and (iii) low CHD risk. Subgroup analyses of the high-risk group were also constructed for individuals who had T2DM and individuals who had a prior history of CVD event. An additional respondent group was identified as those who completed the baseline survey and the two follow-up surveys and had a new CVD event during the 2-year follow up.

Coronary heart disease risk was defined based on NCEP ATP III risk categories using disease status and risk factor counts (4,8). High CHD risk was defined as self-reported diagnosis of heart disease, narrow or blocked arteries/carotid artery disease, stroke or T2DM. For the high CHD risk group, two subgroups were also defined: (i) respondents with T2DM who may also have CVD, and (ii) respondents with major CVD events who may also have T2DM. Moderate to moderately high CHD risk was defined as respondents reporting ≥ 2 of the following risk factors: (i) men > 45 years or women > 55 years of age, (ii) reported diagnosis of low high-density lipoprotein cholesterol, (iii) reported diagnosis of high blood pressure/hypertension, (iv) current smoker and (v) family history of heart disease, narrow or blocked arteries, stroke or diabetes. The lower CHD risk group included respondents with 0–1 of the above risk factors.

T2DM was defined as a self-report of having been ‘told by a doctor, nurse, or other healthcare professional that you have type 2 diabetes.’ CVD event was defined as self-report of heart attack, stroke, heart bypass surgery or angioplasty. If a CVD event was reported at the baseline survey, then the respondent was classified as having a prior CVD event. A new CVD event was defined as no CVD event reported at baseline but a reported event during the 2 years of annual follow-up surveys.

### Therapy assessment

Respondents reported the name of each medication currently prescribed to them. They were instructed to refer to their medication labels for accurate reporting. Lipid-lowering medications included monotherapy and combination therapy of statins, fibric acid derivatives, bile acid sequestrants and cholesterol absorption inhibitors. Prescription antiplatelet and anticoagulant agents included clopidogrel, ticlopidine, cilostazol, dipyridamole, warfarin and low-molecular-weight heparins. For aspirin use, respondents who indicated that they took aspirin every day were considered daily users. Daily aspirin use was examined separately as well as included with the prescription antiplatelet and anticoagulant agents. Antihypertensive medications included ACEIs, ARBs and beta-blockers. Diuretics and calcium channel blockers were not included in the analysis of antihypertensive medications because they are prescribed for several different conditions.

### Statistical analyses

Cross-sectional analysis of CVD medication use (yes/no) was conducted for the baseline respondents. Longitudinal analysis of CVD medication use among respondents with a new CVD event during 2 years of follow up was conducted for those respondents who completed the baseline survey and the two follow-up surveys. The proportion of respondents with a new CVD event who reported drug treatment was computed for: (i) use before and after CVD event, (ii) use after event only, (iii) use before event only, and (iv) no use before or after event. Bivariate analyses included *t*-tests and χ^2^-tests for assessing differences among groups. Logistic regression analyses assessed the likelihood of statin treatment among baseline CHD risk groups, adjusting for age, gender and geographical region. Statistical significance was set *a priori* at p < 0.05.

## Results

### Baseline utilisation

Among baseline survey respondents, 7510 were high CHD risk, 4823 were moderate risk and 5307 were low risk. Low-risk individuals were significantly younger and more likely to be women and had higher education and income than the other groups (p < 0.001) (Table 1).

**Table 1:** Demographic characteristics of SHIELD respondents by CHD risk group

**Characteristics**	**High CHD risk (*n* = 7510)**	**Moderate CHD risk (*n* = 4823)**	**Low CHD risk (*n* = 5307)**
Age, years, mean	60.4	57.2	43.5*
Women, %	56	60	70*
Race, % white	86	88	88
Education, % with some college or higher	64	68	75*
Income, %≥ $40,000/year	47	56	64*
**Geographic region**, %			
Northeast	19	19	19
North Central	24	25	25
South Atlantic	20	20	18
South Central	18	16	16
Mountain	6	6	7
Pacific	13	13	14

* p-value < 0.0001 across risk groups. CHD, coronary heart disease; SHIELD, Study to Help Improve Early evaluation and management of risk factors Leading to Diabetes.

**Figure 1:**
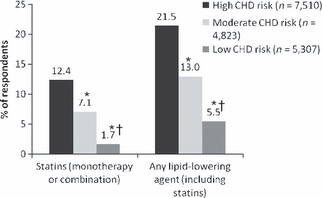
Proportion of SHIELD respondents taking antidyslipidaemia medications. *p < 0.001 for comparison with high-risk group; +p < 0.001 for comparison with moderate-risk group

Utilisation of statin and other lipid-lowering therapy was very low in each group (Figure 1). Significantly more respondents in the high CHD risk group were receiving statins or any lipid-lowering therapy (including statins) compared with moderate-risk or low-risk groups (p < 0.001).

After adjusting for age, gender and geographical region, high CHD risk and moderate CHD risk groups were significantly more likely to have received statin therapy than low-risk respondents (p < 0.001). The high-risk group was four times more likely [odds ratio (OR) = 4.60, 95% confidence interval (CI) = 3.54–5.97] and the moderate risk group was three times more likely (OR = 3.13, 95% CI = 2.45–3.99) to have received statin therapy.

### High risk subgroups

Of the 7510 high CHD risk respondents, there were 3937 respondents who had T2DM (2827 had T2DM only). There were 3777 respondents who reported a prior major CVD event. Within the high-risk group, significantly more respondents with a prior CVD event received lipid-lowering therapy (25.0%) than respondents with T2DM (19.5%) (p = 0.02). Significantly more T2DM respondents were receiving lipid- lowering therapy than moderate and low CHD risk groups (p < 0.001).

**Figure 2:**
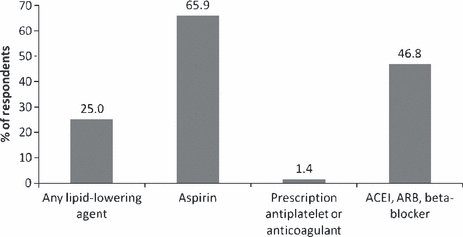
Proportion of respondents with prior CVD event taking CVD medications. ACEI, angiotensin-converting enzyme inhibitor; ARB, angiotensin II receptor blocker; CVD, cardiovascular disease

In examining the use of antiplatelet and antihypertensive therapy among respondents with a prior CVD event, a small proportion of these respondents were taking the drug therapies recommended in the AHA/ACC guidelines for secondary prevention (Figure 2). Approximately 66% of the respondents reported daily aspirin use. For respondents who reported having an MI or stroke at the baseline survey, an additional 1.4% were taking prescription antiplatelet or anticoagulant therapy at the time of the survey. Less than 50% of the prior CVD event group was receiving antihypertensive or dyslipidaemia therapy.

After adjusting for age, gender and geographical region, moderate and low CHD risk groups were significantly less likely to receive statin therapy than T2DM respondents (p < 0.001). The moderate CHD risk group was 28% less likely (OR = 0.72, 95%CI = 0.61–0.86) and the low-risk group was 79% less likely (OR = 0.21, 95% CI = 0.16–0.27) to have received statin therapy. The high CHD risk group (OR = 2.1, 95% CI = 1.7–2.6) and prior CVD event group (OR = 1.25, 95% CI = 1.1–1.5) were significantly more likely to receive statin therapy than T2DM respondents, after adjusting for age, gender and geographical region (p < 0.01).

### Longitudinal utilisation

SHIELD respondents who completed the baseline and two subsequent annual surveys (*n* = 9497) were evaluated to determine the utilisation of CVD medications among those who had an incident CVD event (MI, stroke or revascularisation) during the first 2 years of follow up. Examining this group allowed for closer proximity of medication use to the time of the CVD event occurrence. A total of 953 respondents (10%) reported a new CVD event over the 2 years of follow up, 6% (*n* = 572) in the first year and 4% (*n* = 381) in the second year. Among those who experienced an incident CVD event, 64% were women and 85% white, with a mean age of 59.7 years; 65% had some college education or higher and 31% had T2DM.

There were 1151 new CVD events among the 953 respondents; 198 individuals reported two new events in the 2 years of follow up. MI was the most frequent new CVD event (*n* = 650, 56.5% of events). There were 342 (29.7%) angioplasty or heart bypass surgeries and 159 (13.8%) strokes over the 2 years.

**Figure 3:**
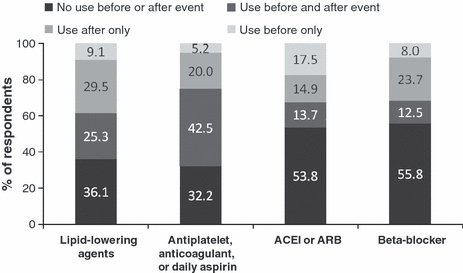
Utilisation of CVD medications before or after the CVD event among respondents with a new CVD event. ACEI, angiotensin-converting enzyme inhibitor; ARB, angiotensin II receptor blocker; CVD, cardiovascular disease

Approximately 36% of respondents with incident CVD events reported not taking lipid-lowering therapy before or after the CVD event. Thirty per cent of incident CVD individuals started lipid-lowering therapy after the event and 9% did not continue with therapy after their event (Figure 3). Antiplatelet, anticoagulant therapy or daily aspirin was not used by 32% of respondents at any point before or after the incident CVD event. An additional 20% of respondents started antithrombotic therapy after their CVD event, while 5% used such therapy only before their incident CVD event. For antihypertensive therapy, approximately 54% of respondents with incident CVD events did not use an ACEI or ARB before or after their CVD event, and utilisation did not increase substantially after the event (15%). Beta-blockers were not used by 56% of respondents before or after their CVD event.

**Figure 4:**
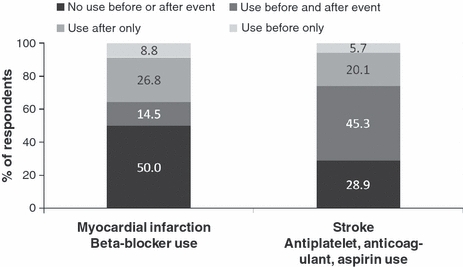
Utilisation of medications by event type among respondents with a new CVD event

To assess whether specific guideline recommendations were being adopted in clinical practice, medication use was examined by CVD event type. AHA/ACC guidelines recommended beta-blockers for individuals with MI (2). About 50% of respondents with a new MI (*n* = 650) did not take a beta-blocker either before or after their MI, and 8.8% used a beta-blocker before their MI (Figure 4). Among new stroke respondents (*n* = 159), 45% were using antiplatelet, anticoagulant or daily aspirin therapy before and after their stroke (Figure 4). An additional 20% of new stroke respondents started antithrombotic therapy after their stroke.

## Discussion

AHA/ACC guidelines (2) recommend lipid-lowering therapy for the prevention of CVD, yet 75% of respondents with a prior MI or stroke, 80% of T2DM respondents, and > 33% of respondents with an incident CVD event report not receiving lipid-lowering therapy. This gap in utilisation of lipid-lowering therapy also existed for high and moderate CHD risk respondents who, by definition, have CHD (high risk) or hypertension and other risk factors (moderate risk). The guidelines also recommend antiplatelet therapy for treatment and prevention of MI and stroke. More than 50% of respondents with a prior CVD event did not receive antiplatelet, anticoagulant or antihypertensive therapy, as recommended in the guidelines. Daily aspirin use was not adopted as a preventive measure by 34% of respondents with a prior CVD event. Additionally, 32% of respondents with an incident CVD event did not take an antiplatelet or anticoagulant agent or daily aspirin, and > 50% did not take an antihypertensive agent (i.e. ACEI, ARB, or beta-blocker) either before or after their event. Preventive drug therapy for MI (beta-blocker) was not taken by 50% of respondents with an incident MI. Antithrombotic therapy utilisation was higher among respondents with incident stroke, with 45% of respondents taking this therapy before and after their stroke and another 20% starting therapy after their stroke.

These findings indicate a gap in preventive care for these at-risk groups. It is possible that the guidelines have not been translated into practice. Physicians may be unaware of the guideline recommendations, or if aware, they may not have adopted the recommendations for all of their at-risk patients. Alternatively, at-risk individuals may have chosen not to initiate or to stop drug therapy possibly because they did not understand their risk and/or the underlying disease process, or because they were concerned about medication-related adverse effects. With a large proportion of at-risk individuals not receiving preventive drug therapy, these study findings are a call to action. Awareness of the guideline recommendations must be raised among physicians to increase their adoption. Individuals at risk, including those with prior or incident CVD event, must be motivated to adopt preventive measures recommended by their physicians for reducing CVD.

This study has several limitations that should be considered in interpreting the results. Respondents were not asked the reason why they were taking aspirin daily; thus, their aspirin use may have been related to other chronic conditions such as arthritis or headaches. Among respondents with a prior CVD event at baseline, information on when the CVD event occurred prior to the survey was not assessed. Thus, individuals with an MI or stroke years ago may have received antiplatelet or anticoagulant therapy around the time of their event but stopped therapy before the baseline survey was administered. The true clinical indication for each drug therapy class could not be assessed because blood pressure and cholesterol levels were not captured in the SHIELD survey. Thus, not all respondents may be candidates for these drug therapies and contraindications or intolerance of certain drugs could not be assessed. Household panels, like the SHIELD study, tend to under-represent the very wealthy and very poor segments of the population and do not include military or institutionalised individuals.

## Conclusions

Based on the study findings, treatment guidelines have not been translated into practice for many respondents in each risk group, including those with prior or incident CVD events. There remains opportunity for significant improvement in raising awareness among physicians to put the recommended therapy guidelines into practice and in motivating at-risk individuals to seek preventive measures for reducing CV disease, especially among respondents with MI. Novel education programmes may be required to increase the adoption of therapy guidelines among clinicians and their at-risk patients.

## References

[1] American Heart Association (2007). Heart Disease and Stroke Statistics – 2007 Update.

[2] Smith SC, Allen J, Blair SN (2006). AHA/ACC guidelines for secondary prevention for patients with coronary and other atherosclerotic vascular disease: 2006 update. Circulation.

[3] Mosca L, Appel LJ, Benjamin EJ (2004). Evidence-based guidelines for cardiovascular disease prevention in women. Arterioscler Thromb Vasc Biol.

[4] National Cholesterol Education Program Adult Treatment Panel III (2002). Third Report of the National Cholesterol Education Program (NCEP) Expert Panel on Detection, Evaluation, and Treatment of High Blood Cholesterol in Adults (Adult Treatment Panel III) final report. Circulation.

[5] Bays HE, Bazata DD, Clark NG (2007). Prevalence of self-reported diagnosis of diabetes mellitus and associated risk factors in a national survey in the US population: SHIELD (Study to Help Improve Early evaluation and management of risk factors Leading to Diabetes). BMC Public Health.

[6] Clark NG, Fox KM, Grandy S, for the SHIELD Study Group (2007). Symptoms of diabetes and their association with the risk and presence of diabetes. Diabetes Care.

[7] US Census Bureau (2003). Annual Supplement to the Current Population Survey: Census Bureau Resident Population Estimates of the United States.

[8] Grundy SM, Cleeman JI, Merz CNB (2004). Implications of recent clinical trials for the National Cholesterol Education Program Adult Treatment Panel III guidelines. Circulation.

